# Exploring the Lived Experience of Acne in the United States and the United Kingdom: Social Media Analysis

**DOI:** 10.2196/91126

**Published:** 2026-06-23

**Authors:** John S Barbieri, Valentine Delore, Isabelle Vieillard, Sandrine Samson, Esteban Puentes, Joelle Malaab, Melanie Sambrès, Manissa Talmatkadi, Stéphane Schück

**Affiliations:** 1 Department of Dermatology, Brigham and Women's Hospital, Harvard Medical School, Boston, MA, United States; 2 Global Medical, Vaccines, Sanofi, Lyon, France; 3 Kap Code, 4 rue de Cléry, Paris, 75002, France, 33 0764853053

**Keywords:** acne, dermatology, social media listening, natural language processing, machine learning, infodemiology

## Abstract

**Background:**

Acne is a chronic skin condition that primarily affects adolescents and young adults but can persist into adulthood. It can have repercussions on physical and mental health, self-esteem, and body image. The increasing use of social media for health information and peer support offers an opportunity to explore real-life experiences with acne.

**Objective:**

This study aims to analyze social media messages from users in the United States and the United Kingdom using artificial intelligence to assess the impact of acne on quality of life (QoL), identify discussion topics, and explore unmet needs.

**Methods:**

The data were extracted from public platforms using a query containing the word “acne” between January 1 and December 31, 2024. Data cleaning and filtering were performed using natural language processing, machine learning methods, and algorithms. Biterm topic modeling was used to identify the main discussion topics, and QoL impact was assessed using a deep learning algorithm adapted from the EuroQol 5-Dimension Questionnaire or the 36-Item Short Form Health Survey. Unmet needs were identified through manual annotation using the saturation method.

**Results:**

A total of 646,809 messages posted by 432,234 users were identified. The main topics included skincare routines and product recommendations (n=154,907, 23.9%), acne scars (n=135,643, 21%), and general treatment information (n=97,177, 15%). Engagement varied across topics and platforms. On Instagram, dietary and nutritional strategies (0.16%, SD 6.36%) showed the highest mean engagement, followed by skincare routines and product recommendations (0.11%, SD 4.81%). In general, engagement scores were higher in the United Kingdom compared to the United States across all topics. On TikTok, content about makeup and acne had the highest mean engagement score (3.03%, SD 92.65%). Overall, 52.9% (228,613/432,234) of the users expressed at least 1 QoL impact, most frequently related to signs and symptoms (175,604/228,613, 76.8%), social functioning (n=149,234, 65.3%), mental health (n=107,155, 46.9%), and cost (n=62,008, 27.1%). Of 3200 annotated messages, 582 contained unmet needs, including effective solutions for hormonal acne (111/582, 19.1%), clarity in identifying acne triggers (n=84, 14.4%), treatment guidance (n=68, 11.7%), and psychological support (n=68, 11.7%).

**Conclusions:**

This study revealed the significant physical, psychological, social, and financial impact of acne on QoL and identified several unmet needs. Given the growing role of social media, these findings highlight opportunities for dermatologists and health professionals to educate and engage with the acne community through digital platforms.

## Introduction

Acne is a chronic skin condition that is estimated to affect 230 million people worldwide [1]. Although most prevalent among young adults and adolescents [2], up to 50% of women and 30% of men experience acne that begins or persists into adulthood [3-6].

Acne can have meaningful effects on individuals’ mental health, self-esteem, and body image [7,8], partly due to the stigmatization they may face at school, in the workplace, and in their personal lives [9]. The global burden of acne in terms of disability-adjusted life years surpasses that of psoriasis and melanoma [10].

Acne is primarily caused by four interrelated factors: excess sebum production, follicular hyperkeratinization, overgrowth of *Cutibacterium acnes* bacteria, and inflammation [11-14]. Treatment options include topical and systemic medications, procedures, diet [15], and lifestyle interventions [16].

In recent years, social media has been increasingly used for various purposes. As of 2025, there are 5.24 billion users, accounting for around 64% of the global population [17]. There is growing interest in social media for health-related purposes, including sharing experiences, seeking advice, accessing medical information, supporting health campaigns, and connecting with others through online communities and support groups [18-22]. Especially given challenges in accessing care, individuals with acne may turn to social media as a source of information and treatment advice [23,24]. At the same time, the growing emphasis on image and beauty trends can be concerning, particularly for adolescents and young adults, who may be more vulnerable to these influences [25].

Given the substantial amount of data that these platforms offer, social media provides a unique opportunity to explore users’ experiences and gain insights into how acne affects their daily lives. While prior studies have evaluated the educational quality of content on social media and focused on limited platforms [26,27], little is known about how individuals with acne engage with social media more broadly, including the effects of acne on quality of life (QoL), topics of discussion, engagement with content, and unmet needs. This study aims to fill this gap by using artificial intelligence techniques, including natural language processing (NLP) and machine learning (ML) to analyze social media messages from users in the United States and the United Kingdom and better understand the effects of acne on users’ QoL, identify key discussion topics, and uncover their unmet needs.

## Methods

### Study Design and Data Sources

This observational, retrospective, real-world study analyzed English-language social media posts in the United States and the United Kingdom between January 1 and December 31, 2024. The data were extracted using the data broker Brandwatch (Cision Ltd.) [28] for all sources except X (formerly Twitter), Instagram, and TikTok, which were extracted via the data broker Visibrain [29]. To perform the extraction, Brandwatch scanned publicly available sources across the internet and retrieved messages that contained the word “acne.” In this approach, platforms and websites are not preselected; rather, the data broker automatically identifies relevant content from publicly accessible sources. These sources include public websites such as online forums, blogs, and health-related discussion platforms.

### Data Processing

Messages were collected using a query consisting of the word “acne” along with their metadata (eg, author and publication date) and compiled into Microsoft Excel. This keyword-based approach was selected to maximize specificity and retrieve messages explicitly discussing acne-related experiences. However, it may have excluded posts referring to acne indirectly, through slang, misspellings, abbreviations, or subtype-specific terminology without explicit mention of the term “acne.”

Geolocation was performed using Brandwatch and Visibrain and verified via a lexical analysis based on country-specific spelling variations (eg, “color” vs “colour”). Thus, 2 distinct datasets were created: 1 for the United States and 1 for the United Kingdom.

The data were cleaned by removing posts with ≤5 words or more than 10,000 characters to optimize text processing. Duplicates, non-English content, and irrelevant or unreliable sources (eg, advertising websites, forums about cars, or animals) were also removed. A supervised ML algorithm using Extreme Gradient Boosting (XGBoost) [30] classifiers (*F*_1_-score: 87%) was applied to identify posts from individuals directly affected by acne.

Following these processing steps, the final “clean” dataset was obtained, consisting exclusively of posts from individuals with acne.

### Ethical Considerations

This study included data from publicly available sources; private groups or web pages were therefore excluded from our data extraction process. We did not seek approval, as users automatically grant their consent for the reuse of their data when they post on public platforms. Furthermore, the results of this study do not contain any identifiable information and are presented in aggregate. Information such as names, usernames or handles, geographic locations, or any other sensitive data was not included. The names of the brands were also removed and replaced with a generic (BRAND) placeholder in the messages.

### Data Analysis

#### Demographics

We identified users’ gender through explicit mentions or inferred using regular expressions (ie, searching usernames for first names, as well as gender-related suffixes and prefixes, and scanning posts for gender-specific vocabulary). Based on these indicators, a scoring system then determined the most likely gender [31].

Age was determined through explicit mentions or predicted using a probabilistic ML model based on features such as syntax, emotional tone, and platform of expression [31].

The remaining cases were classified as “undetermined.”

#### Topics of Discussion

Biterm topic modeling (BTM; via the BTM R package [32]) was applied to all posts. BTM is an NLP, text mining approach that analyzes large volumes of text and clusters similar text based on common topics [32]. The model automatically groups messages into different categories—each representing a specific topic—in descending order of frequency.

In this study, we applied BTM without prior knowledge about the topics that might emerge to ensure an unbiased analysis. As a result, BTM automatically organized the posts into categories, ranked by the frequency with which they appeared in the dataset. Posts could belong to multiple topics. Through human interpretation, these lists of words were used to label the topics, and the associated posts were thoroughly reviewed to ensure correct interpretation.

#### Engagement Rate Analysis by Content Topic and Social Media Platform

For each topic, the engagement rate of every message was calculated using the available engagement parameters, according to the following formula:


$$\text{Engagement rate per message}=\frac{\text{Likes}+\text{Comments on that message}}{\text{Followers at time of posting}}\times 100$$

We then calculated the average engagement rate per topic by computing the mean of all engagement rates from the messages assigned to that topic. Engagement rate data were only available for Instagram and TikTok, so this analysis was limited to these 2 platforms.

#### Impact on QoL

A hybrid NLP approach, adapted from the method developed by Renner et al [33], was used to assess the impact on QoL. The original framework was developed using a manually annotated corpus of 1399 health-related social media messages extracted from online forums discussing multiple diseases and treatments. Messages were manually labeled by trained annotators according to the presence or absence of QoL impact and categorized into predefined QoL dimensions.

The methodology was based on an adaptation of validated QoL questionnaires, the EuroQol 5-Dimension Questionnaire [34] and 36-Item Short Form Health Survey [35], and included 5 dimensions of impact: physical or signs and symptoms, mental health, social functioning, activity-related functioning, and financial burden. In this study, activity-related impacts were integrated within the social functioning dimension.

Consistent with the original methodology, the algorithm incorporates multiple types of features, including lexical fields specific to QoL dimensions, sentiment-related features (eg, expressions of sadness, fear, or negativity), grammatical patterns (eg, pronoun usage, verb tense), and contextual markers of user experiences. The framework also incorporates a rule-based approach using regular expressions (Regex) to identify explicit mentions of QoL-related concepts through predefined linguistic patterns and keywords. During the development of the original framework, several ML algorithms were evaluated, including K-nearest neighbors, support vector machines, random forest, multilayer perceptron, and XGBoost. XGBoost demonstrated the best overall performance and was selected for the final supervised classification models [33]. Separate models were developed for overall QoL impact detection and for each QoL dimension using 5-fold cross-validation and hyperparameter optimization, with the synthetic minority oversampling technique applied to address class imbalance [33].

In this study, the previously developed and validated algorithm was applied to the acne-related dataset. Messages were first classified according to the presence or absence of QoL impact, then assigned to one or more QoL dimensions: signs and symptoms, mental health, social functioning, and cost. The final categorization combined outputs from both the supervised ML models and the Regex-based approach, which enabled the identification of both explicit and implicit QoL impacts.

Classifications were additionally reviewed manually by 2 evaluators (MS and JM) to ensure consistency and relevance in the context of acne-related discussions.

The workflow can be illustrated using the following example:

*My acne makes me feel really anxious about my appearance, and it’s starting to affect my confidence at work and my relationships.*

This message would first be included in the dataset as originating from an individual directly affected by acne. BTM would then assign it to a topic related to self-image and psychological well-being. The QoL algorithm would subsequently classify the message as having an impact on QoL and assign it to the “mental health” and “social functioning” dimensions.

#### Unmet Needs

Two evaluators (MS and JM) annotated messages using an extensive grid consisting of 54 distinct unmet needs. While there are no established guidelines for determining saturation in the context of social media content, this approach is widely accepted in qualitative methodology as a means to determine when further data collection becomes unnecessary [36-38]. To apply this method, successive random samples of messages were annotated until data saturation was reached, defined as ≤2 new unmet needs identified across 2 consecutive samples. Saturation was then confirmed with 2 additional samples.

## Results

### Population and Posts

A total of 1,220,014 messages written by 745,116 internet users discussing acne were retrieved. After data cleaning, 646,809 messages (n=294,786 from the United States; n=352,023 from the United Kingdom) posted by 432,234 users (n=196,911 from the United States; n=235,323 from the United Kingdom) were retained (Multimedia Appendix 1).

A total of 711 sources were included in this study (Multimedia Appendix 2). Over 90% (596,242/646,809) of the messages originated from X (n=304,347, 47.1%), Instagram (n=183,655, 28.4%), and TikTok (n=108,240, 16.7%). Sources contributing individually to less than 0.1% (n≤312) of the dataset, including smaller online forums and community websites, were grouped into the category “Others” (n=28,614, 4.4%). Subsequent analyses were not segmented by source.

Most users self-identified as female (246,373/432,234, 57.0%) compared to male (n=88,176, 20.4%); the rest were undetermined (n=97,685, 22.6%).

Regarding age, 16.0% (69,363/432,234) stated they were under 20 years and 26.5% (n=114,365) were aged 20 to 29 years. Overall, users under 30 represented 42.5% (183,728/432,234) of the dataset, with TikTok users showing the highest proportion in this age group (36,186/79,356, 45.6%). The 30‐ to 39-year category was also prominent (152,728/432,234, 35.3%), particularly on Instagram (45,876/121,688, 37.7%). The mean age was 30.8 (SD 11.24) years (Table 1).

**Table 1.** Characteristics of users posting about acne on the top 3 data sources analyzed (United States and United Kingdom, 2024).

Characteristics	All sources	X/Twitter	Instagram	TikTok	Other
Users, n (%)	432,234 (100)	205,008 (47.4)	121,688 (28.2)	79,356 (18.4)	26,182 (6.0)
Posts, n (%)	646,809 (100)	304,347 (47.1)	183,655 (28.4)	108,240 (16.7)	50,567 (7.8)
User gender, n (%)					
Female	246,373 (57.0)	115,214 (56.2)	71,431 (58.7)	45,154 (56.9)	14,574 (55.7)
Male	88,176 (20.4)	42,642 (20.8)	23,364 (19.2)	16,347 (20.6)	5823 (22.2)
Undetermined	97,685 (22.6)	47,152 (23)	26,893 (22.1)	17,855 (22.5)	5785 (22.1)
User age (y), n (%)					
0‐19	69,363 (16.0)	33,006 (16.1)	18,253 (15.0)	14,284 (18.0)	3819 (14.6)
20-29	114,365 (26.5)	56,582 (27.6)	31,761 (26.1)	21,902 (27.6)	4120 (15.7)
30-39	152,728 (35.3)	70,113 (34.2)	45,876 (37.7)	25,473 (32.1)	11,266 (43.0)
40-49	73,882 (17.1)	34,851 (17.0)	19,713 (16.2)	13,649 (17.2)	5668 (21.6)
>50	13,451 (3.1)	6355 (3.1)	3772 (3.1)	2539 (3.2)	784 (3.0)
Undetermined	8445 (2.0)	4100 (2.0)	2312 (1.9)	1508 (1.9)	525 (2.0)
Age (y), mean (SD)	30.8 (11.24)	30.6 (11.26)	31.0 (11.03)	30.3 (11.45)	32.6 (11.24)

### Topics of Discussion

BTM identified various discussion topics (Table 2).

**Table 2.** List of the main identified topics of discussion about acne among US and UK internet users and their proportion of messages.

Title	US users, n (%)	UK users, n (%)	Total of all messages, n (%)
Discussion about skincare routine and product recommendation	73,492 (24.9)	81,415 (23.1)	154,907 (24.0)
Acne scars	50,162 (17.0)	85,481 (24.3)	135,643 (21.0)
General treatment information	53,675 (18.0)	43,502 (12.4)	97,177 (15.0)
Hormonal acne	43,670 (14.8)	44,158 (12.5)	87,828 (13.6)
Dietary and nutritional strategies	11,780 (4.0)	19,519 (5.5)	31,299 (4.8)
Impact on self-image, self-confidence, and psychological well-being	27,030 (9.2)	NA^a^	27,030 (4.2)
Makeup and acne	6347 (2.2)	16,482 (4.6)	22,829 (3.5)
Discussion about success stories in the treatment navigation	NA	18,643 (5.0)	18,643 (2.9)
Body acne	11,896 (4.0)	2756 (0.8)	14,652 (2.3)
Natural remedies and carcinogens in the treatments	11,083 (2.8)	NA	11,083 (1.7)
Overcoming insecurity beyond the impact on self-image	NA	6747 (1.9)	6747 (1.0)
Total	294,786 (100)	352,023 (100)	646,809 (100)

a “NA” indicates cases where topic modeling did not detect enough messages to generate a distinct topic.

Overall, the most frequently discussed topic centered around skincare routines and product recommendations (154,907/646,809, 24.0%), with users mentioning ingredients such as niacinamide, azelaic acid, and salicylic acid. Acne scars were also frequently discussed (135,643/646,809, 21.0%), particularly in the United Kingdom (85,481/135,643, 24.3%) compared to in the United States (50,162/135,643, 17.0%). Users described the treatments they sought for scarring, including microneedling.

General treatment information represented 15.0% (97,177/646,809) of the messages, including discussions about prescribed treatments such as isotretinoin and birth control and their effects on acne. Hormonal acne was another frequently discussed topic (87,828/646,809, 13.6%), along with dietary and nutritional strategies aimed at improving acne (n=31,299, 4.8%).

Discussions related to the impact of acne on self-image were specific to the United States (27,030/646,809, 4.2%), while topics about success stories in the treatment navigation (n=18,643, 2.9%) and overcoming insecurity beyond self-image (n=6747, 1.0%) were specific to the United Kingdom (Table 2).

### Engagement Rate Analysis by Content Topic and Social Media Platform

Engagement rates help indicate user activity levels across platforms, showing where users are most engaged relative to the audience size.

On Instagram, dietary and nutritional strategies (0.1615%, SD 6.36%) showed the highest mean engagement, followed by skincare routines and product recommendations (0.1108%, SD 4.81%). Hormonal acne (0.0863%, SD 1.76%), success stories in treatment navigation (0.0837%, SD 0.76%), and body acne (0.0809%, SD 2.29%) showed similar engagement levels. In contrast, discussions about natural remedies and carcinogens in treatments generated the lowest mean engagement (0.0417%, SD 0.55%). In general, engagement scores were higher in the United Kingdom compared to the United States across all topics (Table 3).

**Table 3.** Engagement rate analysis on Instagram. $\text{Engagement rate per message}=\frac{\text{Likes}+\text{Comments on that message}}{\text{Followers at time of posting}}\times 100$. Values may exceed one when (1) followers generate multiple interactions (eg, like and comment), (2) followers post more than 1 comment, or (3) content reaches nonfollowers.

Topic	United Kingdom		United States		Total	
	Score (%), mean (SD)	Messages with score available, n	Score (%), mean (SD)	Messages with score available, n	Score (%), mean (SD)	Messages with score available, n
Dietary and nutritional strategy	0.1880 (7.30)	11,350	0.0830 (1.47)	3826	0.1615 (6.36)	15,176
Discussion about skincare routine and product recommendation	0.1300 (5.95)	13,650	0.0760 (1.00)	7519	0.1108 (4.81)	21,169
Hormonal acne	0.1080 (2.08)	1834	0.0350 (0.37)	778	0.0863 (1.76)	2612
Discussion about success stories in the treatment navigation	0.0837 (0.76)	1809	NA^a^	NA	0.0837 (0.76)	1809
Body acne	0.1010 (4.24)	1596	0.0728 (0.37)	3958	0.0809 (2.29)	5554
Overcoming insecurity beyond the impact on self-image	0.0788 (1.28)	2737	0.0540 (0.55)	1019	0.0721 (1.12)	3756
General treatment information	0.0786 (3.07)	15,582	0.0350 (0.34)	3607	0.0704 (2.77)	19,189
Acne scars	0.0667 (1.85)	9084	0.0646 (0.58)	3829	0.0661 (1.58)	12,913
Makeup and acne	0.0638 (0.68)	4392	0.0548 (0.31)	1652	0.0613 (0.60)	6044
Impact on self-image, self-confidence, and psychological well-being	NA	NA	0.0560 (0.55)	1119	0.0560 (0.55)	1119
Natural remedies and carcinogens in the treatments	NA	NA	0.0417 (0.55)	2455	0.0417 (0.55)	2455

a NA: not applicable; the topic was not identified in the corresponding subgroup.

According to the 2024 Social Media Industry Benchmark Report by Rival IQ, the median Instagram engagement rate across all industries is 0.43% [39]. As for the health and beauty industry, the “Engagement Rate per Post” is listed as 0.07% [40].

On TikTok, content about makeup and acne had the highest mean engagement score (3.0337%, SD 92.65%). Discussions about overcoming insecurity beyond the impact on self-image also generated strong engagement (1.1801%, SD 12.82%), followed by body acne (0.8977%, SD 16.07%) and general treatment information (0.8794%, SD 17.13%). Dietary and nutritional strategies (0.8438%, SD 11.22%) and acne scars (0.6198%, SD 7.17%) showed moderate engagement levels, whereas hormonal acne had the lowest mean engagement (0.2570%, SD 2.12%; Table 4).

**Table 4.** Engagement rate analysis on TikTok. $\text{Engagement rate per Message}=\frac{\text{Likes}+\text{Comments on that message}}{\text{Followers at time of posting}}\times 100$. Values may exceed one when (1) followers generate multiple interactions (eg, like and comment), (2) followers post more than 1 comment, or (3) content reaches nonfollowers.

Topic	United Kingdom		United States		Total	
	Score (%), mean (SD)	Messages with score available, n	Score (%), mean (SD)	Messages with score available, n	Score (%), mean (SD)	Messages with score available, n
Makeup and acne	0.8140 (16.09)	1557	9.4696 (180.85)	537	3.0337 (92.65)	2094
Overcoming insecurity beyond the impact on self-image	1.4120 (12.76)	382	0.5650 (13.02)	144	1.1801 (12.82)	526
Body acne	1.0230 (19.56)	1094	0.7090 (8.41)	726	0.8977 (16.07)	1820
General treatment information	0.5350 (5.07)	4159	1.5450 (28.46)	2152	0.8794 (17.13)	6311
Dietary and nutritional strategy	0.5010 (3.8)	1848	2.3100 (24.55)	432	0.8438 (11.22)	2280
Acne scars	0.5250 (5.6)	2440	0.8890 (10.47)	859	0.6198 (7.17)	3299
Discussion about skincare routine and product recommendation	0.3780 (5.15)	6479	0.8540 (12.91)	3495	0.5448 (8.70)	9974
Impact on self-image, self-confidence, and psychological well-being	NA^a^	NA	0.4850 (2.22)	124	0.4850 (2.22)	124
Natural remedies and carcinogens in the treatments	NA	NA	0.2889 (1.42)	319	0.2889 (1.42)	319
Discussion about success stories in the treatment navigation	0.269 (1.72)	423	NA	NA	0.2690 (1.72)	423
Hormonal acne	0.337 (2.51)	808	0.0660 (0.33)	338	0.2570 (2.12)	1146

a NA: not applicable; the topic was not identified in the corresponding subgroup.

According to Rival IQ’s TikTok Benchmark Report, the median engagement rate per follower is 2.6% [41].

### Impact on QoL

In our study, 52.9% (228,613/432,234) of the users expressed at least 1 impact on their QoL. The most frequently cited domains were signs and symptoms (175,604/228,613, 76.8%), followed by social functioning (n=149,234, 65.3%), mental health (n=107,155, 46.9%), and cost (n=62,008, 27.1%; Multimedia Appendix 3).

Among users describing signs and symptoms, 27.7% (48,671/175,604) mentioned pain and itching, while 21.5% (n=37,755) reported scarring and hyperpigmentation. Pimples and redness were also mentioned (n=16,682, 9.5%).

Regarding social functioning, 48.6% (72,528/149,234) reported adapting their skincare routines to alleviate symptoms, while 48.0% (n=71,632) expressed that acne interfered with their professional or school life. Interference with relationships was also reported by 28.6% (n=42,681).

Mental health impacts included feelings of depression and anxiety linked to negative self-image (55,721/107,155, 52%), as well as fear of recurrence (n=6536, 6.1%) and loneliness (n=2786, 2.6%).

The cost of managing acne was also highlighted as a major concern, particularly the cost of treatments and skincare products (20,835/62,008, 33.6%). Users often searched for more affordable treatments (15,254/62,008, 24.6%) or for ones covered by their insurance (n=5271, 8.5%).

### Unmet Needs

A total of 3200 messages were annotated (n=1600 messages per country). Among these, 272 (17.0%) messages in the US dataset and 310 (19.4%) messages in the UK dataset expressed at least 1 unmet need.

The most frequently expressed needs included effective solutions for hormonal acne (111/582, 19.1%), especially treatments considered safe during pregnancy. Users also expressed a need for clarity in identifying acne triggers (84/582, 14.4%). Reported triggers included diet, makeup products, skincare routines, treatments, sun exposure, weather, stress, hormones, sweat, and fitness-related supplements.

Another commonly expressed need involved support and feedback on treatment experiences (68/582, 11.7%), reflecting a perceived lack of reliable information. Many individuals turned to peers or online “experts” for recommendations and opinions on treatments and skincare products.

Psychological support was another mentioned unmet need (68/582, 11.7%), particularly in relation to negative self-esteem, shame, frustration, and social isolation. Additional unmet needs included improved management strategies for acne scars (n=59, 10.1%), clearer guidance throughout the acne care pathway (n=48, 8.2%), and strategies to manage acne triggered by diet (n=33, 5.7%).

Users also expressed a need for more information about specific treatments, mainly isotretinoin, retinoids, and antibiotics (28/582, 4.8%), as well as general advice on managing acne (n=25, 4.3%). Finally, some users mentioned the need for support in managing the financial burden of treatments (n=16, 2.7%; Table 5).

**Table 5.** Top 10 unmet needs related to acne in messages posted in the United States and the United Kingdom in 2024.

Serial number	Needs	United States, n (%)	United Kingdom, n (%)	Total of all messages containing unmet need, n (%)
1	Need for effective solutions for hormonal acne	69 (25.4)	42 (13.5)	111 (19.1)
2	Need for clarity in identifying acne triggers	44 (16.2)	40 (12.9)	84 (14.4)
3	Need for support and feedback on treatment experiences	29 (10.7)	39 (12.6)	68 (11.7)
4	Need for psychological support for acne-related low self-esteem	38 (14)	30 (9.7)	68 (11.7)
5	Need for better management strategies for acne scars	24 (8.8)	35 (11.3)	59 (10.1)
6	Need for clearer guidance through the acne care pathway	17 (6.3)	31 (10.0)	48 (8.2)
7	Need for strategies to manage acne triggered by diet	14 (5.1)	19 (6.1)	33 (5.7)
8	Need for information on specific treatments	8 (2.9)	20 (6.5)	28 (4.8)
9	Need for general advice on acne management	8 (2.9)	17 (5.5)	25 (4.3)
10	Need for support in managing the financial burden of treatments	4 (1.5)	12 (3.9)	16 (2.7)

## Discussion

### Principal Findings

Our findings highlighted the diverse ways in which individuals with acne engage with social media, from January 1 to December 31, 2024. Over half of the posts expressed the effects of acne on QoL, with users describing their symptoms, mental health, and social functioning. These results are consistent with previous research highlighting the negative impact of the condition [42-44]. Consistent with prior survey work, our study identified psychosocial effects including feelings of depression, anxiety, shame, and social isolation [45-48]. Notably, an impact on QoL was also reported among family members of individuals with acne [49]. This may also reflect that those with acne are often younger and may still be living with their family, and is aligned with similar findings that have been observed among those with atopic dermatitis [50].

Most of the retrieved data came from X, Instagram, and TikTok, highlighting these platforms as key spaces where people discuss acne. Users were predominantly female, which aligns with previous studies [51,52] and could reflect that women are more affected by acne across the lifespan or more likely to engage in discussions about their skin on digital platforms.

Users frequently discussed topics such as scarring and hormonal acne, areas that have been less emphasized in previous research. Discussions also included skincare product recommendations and dietary and lifestyle interventions. Users additionally described important unmet needs in these areas, including the need for better psychological support. This aligns with results from another social media study on dermatology, where discussions centered around exchanging tips and advice [53]. However, as social media content on these topics can often be poor or misleading [27], these findings highlight an opportunity for dermatologists and other health professionals to create high-quality, evidence-based educational content on these topics for those with acne. They also support the need for additional research on nonpharmacologic management strategies for acne (eg, diet), treatment for sequelae such as scarring and dyspigmentation, and better management of comorbidities, such as depression and anxiety.

Interestingly, the analysis of engagement rates showed that the topics most frequently discussed were not necessarily those that attracted the most interactions. This suggests that users are more interested in seeking solutions than sharing about their condition [26]. The analysis of engagement rates per platform provided new insights into how users interact with acne-related information depending on the social media platform. On Instagram, messages related to nutrition and skincare showed the highest mean engagement scores. This finding was reported in another acne-related social media study [24], although that work relied only on message volume rather than engagement rates. On TikTok, engagement was higher for content about makeup, which shows a strong interest in finding suitable products for sensitive or breakout-prone skin, as cosmetics have been associated with worsening symptoms [54]. Notably, the topic promoting positive body image received a high engagement rate. Despite the well-documented negative impact of acne on body image and self-esteem [43,55,56], our findings reveal that there may be a growing shift toward self-acceptance. A similar theme was reported in previous research analyzing acne content on TikTok [57], although that analysis was based solely on view counts rather than multimetric engagement used in our study.

To our knowledge, this is the first study to combine a multiplatform and multicountry social media analysis of acne discussions in the United States and the United Kingdom. This study enabled the identification of subtle cross-country differences in frequently discussed topics, their engagement rates per platform, and unmet needs related to acne. These findings provide valuable insights, although they should be interpreted in light of the fact that the study population reflects individuals who publicly discuss acne on social media rather than the general acne population. In addition, differences in platform usage, communication styles, and cultural expressions between countries may have influenced the observed patterns.

### Limitations

This study includes limitations that are common in social media research. The data were derived from publicly available sources; private sources, such as closed Facebook groups and WhatsApp, were thus excluded. These environments may include individuals with different characteristics, including more vulnerable populations or those less likely to share their experiences publicly, which may introduce selection bias. In addition, social media data are based on user-provided information, which may be subject to recall bias, subjective interpretation, and incomplete context. User characteristics, including demographic and clinical information, could not be verified, which should be considered when interpreting the findings and cross-country comparisons. Additionally, individuals posting on social media may belong to certain socioeconomic backgrounds and possess varying literacy capacities, which may affect the representativeness of our findings.

This study was restricted to the United States and the United Kingdom to ensure language consistency and comparability of social media content, as the methodology relies on language-specific features such as lexical fields, sentiment expressions, and grammatical patterns. However, linguistic variations, cultural norms, and differences in how individuals express emotions, symptoms, and social experiences online may influence how QoL impacts are detected and interpreted across populations. In addition, acne-related terminology, slang, and communication styles may differ between countries and languages. Platform availability and usage may also vary across countries, which may potentially influence both the type of users represented and the nature of the content shared. As a result, the transferability of this approach to other countries may require adaptation and validation in different linguistic and cultural contexts. Moreover, it is worth noting that relevant posts might have been mistakenly eliminated during the filtering process. To reduce background noise, we applied threshold values similar to our previous work [58-60], which may have limited the natural processing analysis. Despite these limitations, this study provides valuable insights into the impact of acne on individuals’ lives.

### Conclusions

This study found that acne can have diverse and meaningful effects on QoL. Reports of pain, impaired social functioning, depression, anxiety, and the cost of treatments were commonly described in users’ messages. Important unmet needs were identified, including nonpharmacologic management strategies for acne (eg, diet), treatment for sequelae such as scarring and dyspigmentation, and management of depression and anxiety. Given the growing role of social media, these findings highlight opportunities for dermatologists and health professionals to educate and engage with the acne community through social media. Future research should explore how digital interventions and education campaigns can better address these unmet needs and reduce the psychosocial burden of acne.

## Supplementary material

10.2196/91126 Multimedia Appendix 1 Flowchart of the data cleaning and sample selection processes, showing the number of messages (n) and users discussing acne in 2024.

10.2196/91126 Multimedia Appendix 2 Complete list of sources.

10.2196/91126 Multimedia Appendix 3 Proportions of US and UK internet users reporting the impact of acne on their quality of life on social media in 2024.

## Abbreviations

BTM: biterm topic model
ML: machine learning
NLP: natural language processing
QoL: quality of life
XGBoost: Extreme Gradient Boosting
