# Supplementary figures and images for: Exploring the Lived Experience of Acne in the United States and the United Kingdom: Social Media Analysis
Source: JMIR Dermatol. 2026 Jun 23;9:e91126. doi: 10.2196/91126 (PMC13290109; doi:10.2196/91126)

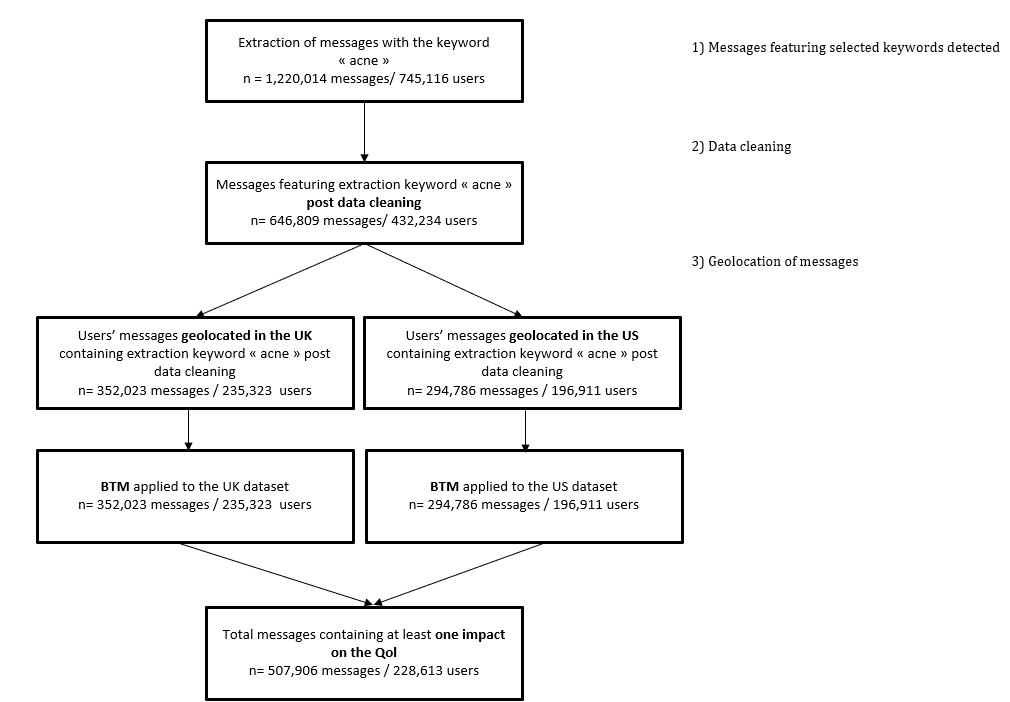

Supplement: Multimedia Appendix 1 [file derma-v9-e91126-s001.docx]
