# Supplementary material for: Exploring the Lived Experience of Acne in the United States and the United Kingdom: Social Media Analysis
Source: JMIR Dermatol. 2026 Jun 23;9:e91126. doi: 10.2196/91126 (PMC13290109; doi:10.2196/91126)
Supplement: Multimedia Appendix 2 [file derma-v9-e91126-s002.docx]

| **Forum/Social Media** | **n posts** | **% posts** |
| --- | --- | --- |
| X | 304347 | 47.1% |
| Instagram | 183655 | 28.4% |
| Tiktok | 108240 | 16.7% |
| 4channel.org | 9409 | 1.5% |
| whattoexpect.com | 9406 | 1.5% |
| sephora.com | 1036 | 0.2% |
| lipstickalley.com | 683 | 0.1% |
| babycenter.com | 574 | 0.1% |
| makeupalley.com | 517 | 0.1% |
| worldstar.com | 328 | 0.1% |
| Others | 28,614 | 4.4% |
