# Supplementary material for: Exploring the Lived Experience of Acne in the United States and the United Kingdom: Social Media Analysis
Source: JMIR Dermatol. 2026 Jun 23;9:e91126. doi: 10.2196/91126 (PMC13290109; doi:10.2196/91126)
Supplement: Supplementary file 3 [file derma-v9-e91126-s003.docx]

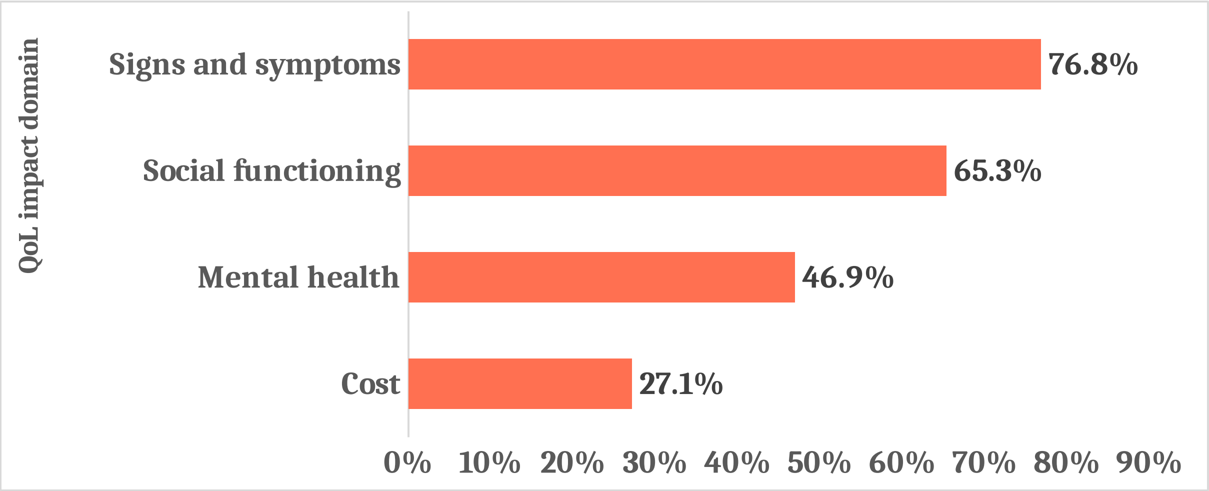
QoL impact domain
Signs and symptoms
Social functioning
Mental health
Cost
76.8%
65.3%
46.9%
27.1%
0%
10%
20%
30%
40%
50%
60%
70%
80%
90%
